# Does long-term coffee intake reduce type 2 diabetes mellitus risk?

**DOI:** 10.1186/1758-5996-1-6

**Published:** 2009-09-16

**Authors:** Gustavo D Pimentel, Juliane CS Zemdegs, Joyce A Theodoro, João F Mota

**Affiliations:** 1 Department of Physiology, Division of Nutrition Physiology, Federal University of Sao Paulo (UNIFESP), Sao Paulo, Brazil; 2 Department of Nutrition, Nutrition and Health Sciences Institute, Campinas, Brazil

## Abstract

This review reports the evidence for a relation between long-term coffee intake and risk of type 2 diabetes mellitus. Numerous epidemiological studies have evaluated this association and, at this moment, at least fourteen out of eighteen cohort studies revealed a substantially lower risk of type 2 diabetes mellitus with frequent coffee intake. Moderate coffee intake (≥4 cups of coffee/d of 150 mL or ≥400 mg of caffeine/d) has generally been associated with a decrease in the risk of type 2 diabetes mellitus. Besides, results of most studies suggest a dose-response relation, with greater reductions in type 2 diabetes mellitus risk with higher levels of coffee consumption. Several mechanisms underlying this protective effect, as well as the coffee components responsible for this association are suggested. Despite positive findings, it is still premature to recommend an increase in coffee consumption as a public health strategy to prevent type 2 diabetes mellitus. More population-based surveys are necessary to clarify the long-term effects of decaffeinated and caffeinated coffee intake on the risk of type 2 diabetes mellitus.

## Introduction

Type 2 diabetes mellitus (DM2) is characterized by insulin resistance and/or abnormal insulin secretion, resulting in a decrease in whole-body glucose disposal. Individuals with chronic hyperglycemia, insulin resistance, and/or DM2 are at greater risk for hypertension, dyslipidemia, and cardiovascular disease [1].

Although genetic factors may play a role in the etiology of DM2 [2], there is now convincing evidence that DM2 is strongly associated with modifiable factors, such as diet. Interestingly, among the several factors present in diet, coffee, one of the most widely consumed non-alcoholic beverages in Western society [3,4], is highlighted as a potent dietary-component associated with reduced risk of several chronic diseases, including DM2 and its complications [5-11]. Coffee is a complex mixture of more than a thousand substances, including caffeine (primary source), phenolic compounds (chlorogenic acid and quinides - primary source), minerals and vitamins (magnesium, potassium, manganese, chromium, niacin), and fibers [12] and several of these coffee constituents have a possible role in glucose metabolism.

The present review provides an overview of the role of long-term coffee intake on the risks of glucose tolerance, insulin sensitivity, and DM2.

### Coffee intake and type 2 diabetes mellitus: a link between cohort and systematic review studies

The association between the coffee intake and the risk of developing DM2 has been examined by several researches. Cohort studies and a systematic review are summarized in Table 1. Data from a prospective study indicated an inverse association between coffee consumption and the risk of DM2 in men independently of race, age or serum concentration of magnesium. Individuals who drank at least seven cups of coffee daily had 50% lower risk to develop DM2 than those who drank two cups or fewer per day [7]. However, this study has not differenced the intake of caffeinated and decaffeinated coffee and didn't evaluate other sources of caffeine.

**Table 1:** Cohort studies of coffee consumption and risk of type 2 diabetes mellitus.

**Reference**	**Experimental Protocol/** **Follow-up (y)**	**Subjects**	**Dose (cups/d)†**	**Results** **Relative Risk (95% Confidence Interval)**
**van Dam & Feskens, 2002 **[7]	Prospective cohort/7	117111 M and W	≤2	1 (reference)
			3-4	0.79 (0.57-1.10)
			5-6	0.73 (0.53-1.01)
			≥7	0.50 (0.35-0.72)
				
**Saremi et al., 2003 **[20]	Prospective cohort/11	2680 M and W	0	1 (reference)
		Pima Indians	1-2	0.92 (0.74-1.13)
			≥3	1.01 (0.82-1.26)
				
**Reunanen et al., 2003 **[19])	Prospective cohort/16	19518 M and W	≤2	1 (reference)
			3-4	1.01 (0.81-1.27)
			5-6	0.98 (0.79-1.21)
			≥7	0.92 (0.73-1.16)
				
**Rosengren et al., 2004 **[10]	Prospective cohort/18	1361 W	≤2	1 (reference)
			3-4	0.55 (0.32-0.95)
			5-6	0.39 (0.20-0.77)
			≥7	0.48 (0.22-1.06)
				
**Salazar-Martinez et al., 2004 **[8]				
-Health Professionals Follow-up Study	Prospective cohort/12	41934 M	0	1 (reference)
			1-3	0.93 (0.80-1.08)
			4-5	0.71 (0.53-0.94)
			≥6	0.46 (0.26-0.82)
				
-Nurses' Health Study	Prospective cohort/18	84276 W	0	1 (reference)
			1-3	0.99 (0.90-1.08)
			4-5	0.70 (0.60-0.82)
			≥6	0.71 (0.56-0.89)
				
**Tuomilehto et al., 2004 **[11]	Prospective cohort/12	14629 M and W	≤2	1 (reference)
			3-4	0.76 (0.57-1.01)
			5-6	0.54 (0.40-0.73)
			7-9	0.55 (0.37-0.81)
			≥10	0.39 (0.24-0.64)
				
**Carlsson et al., 2004 **[9]	Prospective cohort/20	10652 M and W	≤2	1 (reference)
			3-4	0.70 (0.48-1.01)
			5-6	0.71 (0.50-1.01)
			≥7	0.65 (0.44-0.96)
				
**van Dam et al, 2004 **[63]	Cross-sectional and prospective data/6	1312 M and W	5	Cross-sectional: lower fasting insulin concentrations but not with lower fasting glucose concentrations
Hoorn Study				
				Prospective:
			≤2	1 (reference)
			3-4	0.94 (0.56-1.55)
			5-6	0.92 (0.53-1.61)
			≥7	0.69 (0.31-1.51)
				
**van Dam & Hu, 2005 **[21]	Systematic review (9 cohorts)	193473 M and W	≤2	1 (reference)
			4-6	0.72 (0.62-0.83)
			≥6	0.65 (0.54-0.78)
				
**Greenberg et al., 2005**[34]	Prospective cohort/8.4	7006 M and W	2	Caffeinated 0.86 (0.75-0.99)
First National Health and Nutrition			2	Decaffeinated 0.58 (0.34-0.99)
Examination Survey Epidemiologic				Further analysis revealed that the decrease in DM2 risk only applied to those who had lost weight
Follow Up Study				
				
**van Dam et al., 2006 **[13]	Prospective cohort/10	88259 W	0	1 (reference)
Nurses' Health Study II			1	0.87 (0.73-1.03)
			2-3	0.58 (0.49-0.68)
			≥4	0.53 (0.41-0.68)
				
**Iso et al., 2006 **[6]	Retrospective cohort/5	17413 M and W	0	1 (reference)
			1-2	0.93 (0.73-1.19)
			≥3	0.58 (0.37-0.90)
				
**Pereira et al., 2006 **[14]	Prospective/11	28812 W	0	1 (reference)
Iowa Women's Study			1-3	1.01 (0.85-1.19)
			≥6	0.78 (0.61-1.01)
			Decaffeinated	0.67 (0.42-1.08)
			Caffeinated	0.79 (0.59-1.05)
				
**Smith et al., 2006 **[18]	Prospective/8	910 M and W	Never	1 (reference)
Rancho Bernardo			Former	0.36 (0.19-0.68)
			Current	0.38 (0.17-0.87)
				
**Paynter et al., 2006 **[5]	Prospective/12	12204 M and W	≥4	M: 0.77 (0.61-0.98)
ARIC Study				
				
**Schulze et al., 2007 **[64]	Prospective/7	25167 M and W	150 g/d	0.96 (0.93-0.99)
EPIC-Potsdam				
				
**Hamer et al., 2008 **[35]	Prospective/11.7	5823 M and W	0	1 (reference)
Whitehall II Study			<1	0.83 (0.60-1.14)
			2-3	0.85(0.60-1.20)
			>3	0.80(0.54-1.18)
				
**Odegaard et al., 2008 **[65]	Prospective/6	36908 M and W	0	1 (reference)
Singapore Chinese Health Study			1	0.96 (0.86, 1.08)
			2-3	0.90 (0.79, 1.02)
			≥4	0.70 (0.53-0.93)

M: Men; W: Women; †: ~150 mL cup.

Salazar-Martinez et al [8] evaluated the intake of coffee and caffeine from any sources and found an association between coffee intake and the risk of DM2. Besides, this association was found to be more prominent in women than in men and a protective effect of caffeine intake against DM2 was also revealed.

In the Nurses' Health Study II, the researchers observed, after adjustment for several variables, a lower risk of DM2 in women who consumed any dose of coffee when compared to those who did not have this habit. This association was similar in both caffeinated 0.87 (CI: 0.83-0.91), decaffeinated 0.81 (CI: 0.73-0.90) and filtered coffee 0.86 (CI: 0.82-0.90), suggesting that moderate, either caffeinated, decaffeinated or filtered, coffee consumption decreases (13-19%) the risk of DM2 in young and middle-aged women [13].

The 11-year prospective Iowa Women's Health Study, carried out with postmenopausal woman verified that the intake of both types of coffee, caffeinated and decaffeinated, was inversely associated to the risk of DM2 [14]. In accordance to this, the Nurses' Health Study I (1989-1990) revealed a 16% lower concentration of C-peptide in individuals who ingested at least 4 cups of caffeinated or decaffeinated coffee per day, indicating that the chronic consumption of caffeinated/decaffeinated coffee might reduce insulin secretion since it decreases C-peptide secretion, a marker of insulin secretion [15] and reducing insulin secretion is consistent with increased insulin sensitivity. The results from these studies indicate that coffee constituents other than caffeine might have a protective role against DM2.

Additionally, an epidemiological study indicated that coffee processing seems to have an effect in the risk of DM2 and pointed an advantage of the filtered coffee over the boiled one (without filtering) in reducing the risk of DM2 [11]. Since the lipidic substances from coffee grains, namely cafestol and kahweol, are removed in filtered coffee [16,17], it is reasonable to suggest that these substances might act indirectly by increasing the risk of DM2. Moreover, another epidemiological study observed that the protective effect of coffee intake depended on the doses [10] and a prospective study reported that both current and former (~20 ago) coffee consumers had, respectively, 62% and 64% reduction in the risk of DM2 [18].

As verified, not all studies have observed an inverse association between coffee consumption and the risk of DM2. In fact, a Finnish cohort study didn't report this association [19]. In addition, a study in Pima Indians, a population with high prevalence of DM2, didn't find different incidence of DM2 among coffee consumers and who those who never drink coffee [20]. Nevertheless, a systematic review elaborated from nine cohort studies supports the inverse association between coffee consumption and the risk of DM2. The individuals who ingested 4-6 cups per day and those with higher intake (more than 6 cups of coffee per day) had 28% and 35% lower risks of DM2 when compared to the lowest ingestion category (less than 2 cups or none daily) [21].

### Can caffeine reduce the risk of DM2?

Among coffee constituents, caffeine (1, 3, 7 trimethylxanthine) has received more attention due to its physiological and pharmacological properties, mainly regarding its effect on sleep reduction and stimulant properties [22].

Caffeine can be completely absorbed by the stomach and small intestine within 45 minutes after intake and it reaches maximum concentration in the bloodstream in 15-120 minutes [23]. Once absorbed, caffeine is distributed all over the body [24]. In line with this, Biaggioni et al [25] showed linear correlations between the concentrations of caffeine in plasma and brain (r = 0.86) and between concentrations in plasma and kidney (r = 0.91). Besides, Eskenazi [26] demonstrated that caffeine can cross the placenta and be found in the mother's milk.

Caffeine metabolization takes place in the liver, starting by the removal of the methyl 1 and 7 groups in a reaction catalyzed by cytochrome P450, enabling the formation of three methylxanthine groups: paraxantine (84%), theobromine (12%) e theophylline (4%). Each component has a different role in human physiology; in particular, paraxantine increases lypolisis; theobromine stimulates blood vessels dilatation and increases the urine volume; and theophylline controls the glucose metabolism [27].

Blood concentrations of caffeine or its metabolites reflect caffeine intake in the previous hours [28]. However, caffeine intake may not correlate strongly with coffee intake, as it also depends on the intake of other sources of caffeine. Table 2 shows the caffeine/magnesium content of selected food and drink.

**Table 2:** Caffeine and magnesium content of selected food and drinks

**Food or drink**	**Caffeine mg**	**Magnesium mg**
Regular coffee, brewed from grounds*	95	7
Regular coffee, brewed from grounds, decaffeinated*	2	12
Coffee, brewed, espresso*	509	192
Regular instant coffee*	62	7
Decaffeinated instant coffee*	2	12
Carbonated beverage, cola†	29	0
Energy drink†	108.4	11.6
Tea, brewed*	47	7
Milk chocolate bar‡	9	28

Source: U.S. Department of Agriculture Agricultural Research service (2007).

* cup of 237 mL or 8 fl oz

† can of 355 mL or 12 fl oz

‡ bar of 44 g or 1.55 oz

The mean per capita caffeine intake in the Western society is 300 mg/d, essentially consumed from dietary sources such as coffee, tea, cola drinks and chocolate [29]. Data from the National Health and Nutrition Examination Surveys (NHANES III) showed that the American population consumes nearly 236 mg/d of caffeine from coffee and tea [4]. In Brazil, literature about caffeine intake is scarce. A research carried out in Rio de Janeiro city among pregnant women under care at a maternal infant unit found out the caffeine consumption to be 56.2 mg/d, being coffee (~40 mg) the most significant food source, followed by tea (~11 mg) and chocolate powder (~5 mg) [30].

Human studies indicate that caffeine intake of ~500 mg/d does not lead to dehydration or water imbalance [31,32]. Moreover, moderate caffeine intake (~400 mg/d) is not associated with increased risk of hypertension, heart disease, osteoporosis, or high plasma cholesterol [33].

The Canadian Clinical Practice Guidelines [34] reported that for the average adult, a daily caffeine intake of 400-450 mg is not associated with any adverse effects. The recommendation for pregnant women and those who are breastfeeding is reduced to 300 mg/day; and for children, it is limited to their age (Table 3).

**Table 3:** Caffeine recommendation according to age.

**Individuals**	**Recommendation (mg/day)**
Children	-
4-6 years old	45.0
7-9 years old	62.5
10-12 years old	85.0
Adults	400
Pregnant/Breastfeeding women	300

Source: Canadian Clinical Practice Guidelines [34]

Some of the above mentioned researches have examined the association between decaffeinated coffee intake and risk of DM2 [8,13,14,35,36]. Three out of five studies have found significantly positive association between decaffeinated coffee intake and risk of DM2 and in one of these studies decaffeinated coffee tended to be associated with a lower risk of DM2. Besides, Wu et al [15] reported similar associations with lower plasma C-peptide concentrations and the intake of caffeinated and decaffeinated coffee, suggesting that both types of coffee exert a beneficial effect on insulin sensitivity. It follows from this that coffee components other than caffeine may be responsible for these effects.

### Mechanisms underlying the protective effects of coffee intake on DM2

**Figure 1:**
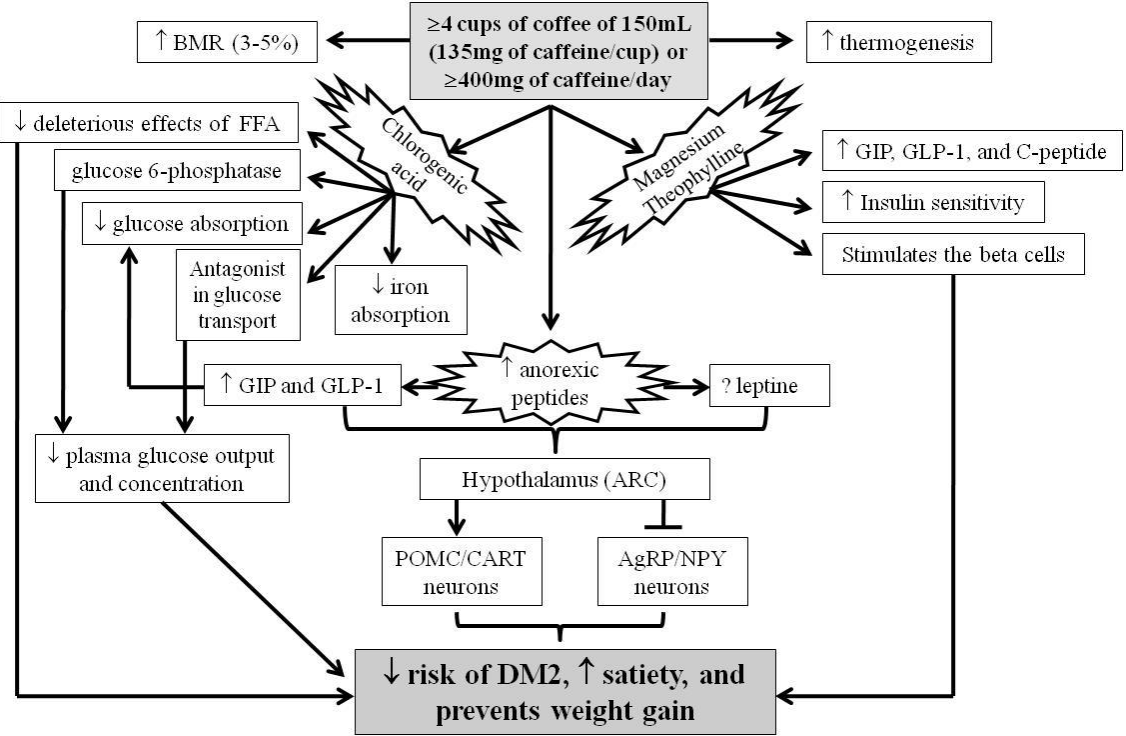
**Mechanisms of action of coffee and your constituents responsible for reduce the risk of the DM2**. BMR: basal metabolic rate, FFA: free fatty acids, ARC: arcuate nucleus, GIP: polypeptide pancreatic, GLP-1: glucagon-like peptide 1, POMC: proopiomelanocortin, CART: cocaine- and amphetamine-regulated Transcript, AgRP: agouti-related protein, NPY: neuropeptide Y.

Up to the moment, several mechanisms of action as well as the precise coffee constituent responsible for the association between coffee intake and DM2 have been proposed. Figure 1 is the summary of studies listed bellow in text.

The hypothesis that coffee consumption lowers the risk of DM2 involves several possible mechanisms as its likely effects on obesity and insulin sensitivity, which are important risk factors for DM2 [1]. In accordance to this, Tagliabue et al [37] proposed that coffee consumption might stimulate thermogenesis. Some studies showed that caffeine intake is inversely associated with body weight gain and satiety. Lopez-Garcia et al [38], in his latest research of a 12-year follow-up assessing men and women showed that individuals who consumed coffee lost more weight than those who did not.

Besides, a randomized, placebo-controlled and double-blind study with overweight and moderately obese men and women noticed that the intake of a high-caffeine diet (~524 mg/d) reduces body weight, fat mass and waist circumference, and increases the satiety, when compared to a low-caffeine diet (~151 mg/d) [39]. Accordingly, Kovacs et al [40] observed that high caffeine consumption (511 mg/d) led to higher satiety than low caffeine intake (149 mg/d).

Additionally, coffee influences the secretion of gastrointestinal peptides such as glucagon-like peptide-1 (GLP-1) and gastric inhibitory polypeptide (GIP), lowering glucose absorption in the small intestine [41,42], and activating central anorexigenic peptides (POMC/CART) as well as inhibiting orexigenic peptides (AgRP/NPY) [43,44]. In accordance to this, McCarty [45] reports a higher GLP-1 production after the intake of drinks containing chlorogenic acid, such as coffee. Another suggested mechanism is the direct stimulation of pancreatic beta cells by caffeine and theophylline [46].

The beneficial effects of coffee's constituents other than caffeine on insulin sensitivity should be considered. Coffee is a major source of the polyphenol chlorogenic acid in the human diet and may affect glucose metabolism by different mechanisms: increasing insulin sensitivity [47]; inhibiting glucose absorption [48]; inhibiting or retarding the action of α-glucosidase [49]; inhibiting glucose transporters at the intestinal stage [50]; reducing or inhibiting glucose-6-phosphatase hydrolysis at the hepatic stage, what may reduce plasma glucose output, leading to reduced plasma glucose concentration [51-54]. Moreover, this acid neutralizes the deleterious effects of free fatty acids over the function of beta cells in insulin-resistant overweight individuals, reducing the risk of DM2 [45]. However, it is important to take into account potential confounding by other foods sources of chlorogenic acid, such as apples [47].

Furthermore, it has been suggested that the inhibition of iron absorption by polyphenol compounds present in coffee might be one of the mechanisms underlying the protective effects of coffee intake on glucose metabolism [55] as evidences points that higher body iron stores are associated with an increased risk for DM2 [56]. In line with this, the induction of iron deficiency in impaired glucose tolerant subjects has improved insulin sensitivity [57].

Each cup (237 mL; 8 fl oz) of regular instant coffee has nearly 7 mg of magnesium (Table 2), a micronutrient involved in glucose homeostasis [58-60]. Preliminary data evidenced an association between low dietary magnesium intake and insulin resistance [61]. Accordingly, low plasma magnesium concentrations were found in the Pima Indians, probably due to their high degree of insulin resistance [62].

## Conclusion

For many years, diet has been noticed as an important modifiable determinant of chronic diseases such as DM2. The association between coffee intake and reduction in the risk of DM2 development is plausible and has been consistently demonstrated in longitudinal studies in diverse populations.

The majority of epidemiological studies, as well as the systematic review about the issue, indicate that the long-term intake of coffee, caffeinated or decaffeinated, can reduce the risk of DM2, being moderate coffee intake (≥4 cups of coffee/d of 150 mL or ≥400 mg of caffeine/d) the disclosed benefic dose. It is noticeable that results of most studies suggest a dose-response relation, with greater reductions in DM2 risk in the higher levels of coffee intake, and that adjusting the associations for potential confounding normally strengthened this inverse association. Even though none of the studies found any negative effects of coffee over the risk of DM2, it is also important to highlight that habitual coffee/caffeine consumption have been related to deleterious effects such as bone loss in elderly postmenopausal women, increases in serum homocysteine and cholesterol and blood pressure, as well as risk of coronary heart disease.

Currently, several substances other than caffeine, e.g. chlorogenic acid and magnesium, have been suggested as responsible for the protective effect of coffee in the risk of DM2. However, since it is difficult to control all confounder's variables and consider individual's behaviors, the precise coffee constituent responsible for this association remains uncertain, as well as the mechanisms underlying the beneficial effects of coffee intake over glucose metabolism.

Although habitual moderate coffee intake seems to be safe and reduce the risk of DM2, referenced researchers [21] in the theme state that it is early to recommend an increase in coffee consumption as a public health strategy for preventing diseases.

## Competing interests

The authors declare that they have no competing interests.

## Authors' contributions

GDP made substantial contributions to conception and design, acquisition of data. He has also been involved in drafting the manuscript and revised it critically as corresponding author. GDP and JCSZ screened all titles and abstracts, assessed the full text papers and checked the extracted study data and participated in the critical revision of include articles and in the writing of the manuscript. JAT and JFM have been involved in developing the electronic-search strategy, contributed in the acquisition of data, and revised the manuscript critically. All authors have made substantial contributions to the analysis and interpretation of include articles. All authors have given final approval of the submitted version.
